# Effect of virgin olive oil as spreadable preparation on atherosclerosis compared to dairy butter in Apoe-deficient mice

**DOI:** 10.1007/s13105-024-01029-8

**Published:** 2024-05-24

**Authors:** Roberto Martínez-Beamonte, Cristina Barranquero, Sonia Gascón, Juan Mariño, Carmen Arnal, Gloria Estopañán, María Jesús Rodriguez-Yoldi, Joaquín Carlos Surra, Olga Martín-Belloso, Isabel Odriozola-Serrano, Israel Orman, Jose Carlos Segovia, Jesús Osada, María Ángeles Navarro

**Affiliations:** 1 Departamento de Bioquímica y Biología Molecular y Celular, Facultad de Veterinaria, Instituto de Investigación Sanitaria de Aragón, Universidad de Zaragoza, 50013 Saragossa, Spain; 2 Instituto Agroalimentario de Aragón, CITA-Universidad de Zaragoza, 50013 Saragossa, Spain; 3 CIBER de Fisiopatología de La Obesidad y Nutrición, Instituto de Salud Carlos III, 28029 Madrid, Spain; 4 Illes Balears, Instituto de Medicina Legal de Las Islas Baleares, E-07003 Palma, Spain; 5 Las Arbequinas de Rosalía, Monesma de San Juan, 22415 Huesca, Spain; 6 Departamento de Patología Animal, Facultad de Veterinaria, Universidad de Zaragoza, 50013 Saragossa, Spain; 7 Centro de Investigación y Tecnología Agroalimentaria de Aragón (CITA), Avda. Montañana 930, 50059 Saragossa, Spain; 8 Departamento de Farmacología , Fisiología y Medicina Legal y Forense, Facultad de Veterinaria, Instituto de Investigación Sanitaria de Aragón, Universidad de Zaragoza, E-50013 Saragossa, Spain; 9 Departamento de Producción Animal y Ciencia de los Alimentos, Instituto de Investigación Sanitaria de Aragón, Escuela Politécnica Superior de Huesca, Universidad de Zaragoza, 50013 Saragossa, Spain; 10 Department of Food Technology, Engineering and Science, University of Lleida, Av. Alcalde Rovira Roure, 191, 25198 Lleida, Spain; 11 Agrotecnio-CERCA Center, Av. Rovira Roure, 191, 25198 Lleida, Spain; 12 Oliberus, Campus Iberus, Zaragoza, Spain; 13 Alimentos Funcionales, Campus Iberus, Zaragoza, Spain; 14 Cell Technology Division. Centro de Investigaciones Energéticas Medioambientales y Tecnológicas (CIEMAT) and Centro de Investigación Biomédica en Red de Enfermedades Raras (CIBERER), CIEMAT/CIBERER, Madrid, Spain; 15 Present Address: Advanced Cell Therapy Unit., Instituto de Investigación Sanitaria Fundación Jiménez Díaz, Madrid, Spain

**Keywords:** EVOO, Coronary artery disease (CAD), Phenols, Apolipoprotein E deficient mice

## Abstract

Olive oil is the main source of lipid energy in the Mediterranean diet and there is strong evidence of its health benefits. The effect of extra virgin olive oil (EVOO) in the form of a preparation of spreadable virgin olive oil (S-VO) on the progression of atheroma plaques was investigated in *Apoe*-deficient mice, a model of accelerated atherosclerosis. Methods: Two isocaloric Western purified diets containing 20% fat, either as S-VO or as dairy butter, were used to feed 28 males and 16 females of two-month-old *Apoe*-deficient mice for 12 weeks. S-VO was prepared by blending more than 75% virgin olive oil with other vegetal natural fat to obtain a solid fat. Plasma total cholesterol, triglycerides and HDL cholesterol were measured. Hepatic lipid droplets were analyzed. Areas of atherosclerotic aortic lesions were quantified in cross-sectional images of the proximal aorta and en face analysis of the whole aorta. Results: Total plasma cholesterol was increased in mice on the butter-supplemented diet in both female and male mice compared to S-VO, and the ratio of TC/HDL-cholesterol was significantly lower in S-VO than in the butter diet, although only in males, and no differences in plasma triglycerides were observed. No significant differences in hepatic lipid droplets were observed between diets in either sex. Aortic lesion areas were significantly higher in mice consuming the butter versus the S-VO diet in both sexes. Conclusion: Extra virgin olive oil prepared in spreadable form maintained the delay in atheroma plaque progression compared to butter.

**Supplementary Information:**

The online version contains supplementary material available at 10.1007/s13105-024-01029-8.

## Introduction

The Mediterranean diet is associated with a low incidence of metabolic diseases and longer life expectancy [17, 22, 45]. Although there are some geographical variations in this dietary pattern, virgin olive oil (VOO) is always the main source of fat [15]. Two landmark studies have shown that the use of extra virgin olive oil (EVOO) as part of a Mediterranean diet resulted in reduced mortality in primary [13] and secondary cardiovascular [11] prevention.

According to food consumption data from the Food and Agriculture Organization (FAO) of the United Nations, most European countries consume more butter than olive oil. Only Spain, Portugal, Italy, Albania, Greece and Luxembourg show the opposite trend [34]. In order to adapt to European consumers’ preferences for cooking with solid fats and to offer them the benefits of using EVOO, a spreadable virgin oil (S-VO) has been prepared by mixing it with other fats to give it a solid consistency similar to butter at room temperature. In this way, the consumption of S-VO can contribute to a healthier diet in these countries, while maintaining the same culinary tradition. To achieve this goal and to validate the biological properties of the new S-VO in pathologies related to Western societies, a dietary intervention was carried out in an animal model that develops atherosclerosis and fatty liver. In fact, the Apoe-deficient mouse has been the subject of research on atherosclerosis and dietary interventions [36]. This model also develops fatty liver [18], more pronounced when a Western diet enriched with 20% fat and 0.15% cholesterol is provided for 12 weeks [19, 31]. Apoe-deficient mice, as a model that develops both pathologies, are used to test the properties of a S-VO compared to butter.

## Material and Methods

### Spreadable virgin olive oil-based preparation (S-VO)

The S-VO was prepared using at least a 75% of EVOO elaborated with olives obtained from olive groves owned by "Las arbequinas de Rosalía" located in Somontano de Barbastro (Huesca, Spain) and collected on an organic regime, with early harvest and milled in the same region to obtain the EVOO. In order to transform EVOO into S-VO, mixtures at low temperature of this EVOO with organic cocoa butter were prepared to obtain the S-VO with the desired texture and consistency, solid at room temperature as butter, and with the maximum amount of EVOO to maximize its biological benefits.

### Animals and Experimental Procedure

*Apoe*-deficient mice on the C57BL/6 J genetic background were purchased from Charles River (Charles River Laboratories, Barcelona, Spain) and bred at the *Centro de Investigación Biomédica de Aragón* (CIBA) in Zaragoza, Spain. To establish groups with similar initial body weight and plasma cholesterol levels, 9- to 10-week-old mice (28 males and 16 females) were weighed, blood samples taken from the facial vein (after a four-hour fast), and their cholesterol levels analyzed. Four groups of *Apoe*-deficient mice were assigned, 2 groups for males and another 2 for females, and housed in sterile filter-top cages in rooms maintained on a 12-h light/12-h dark cycle in the CIBA. All had ad libitum access to food and water. Solid feed intake was monitored weekly by cage, quantifying the difference between the offered and the refused divided by mice/cage to estimate individual daily feed intake, and live body weight was recorded every two weeks. Mouse experiments were performed in accordance with the EU Directive 2010/63 on the protection of animals used for scientific purposes, and the study protocol was approved by the Animal Ethics Committee of the University of Zaragoza with code PI15/07.

**Fig. 1:**
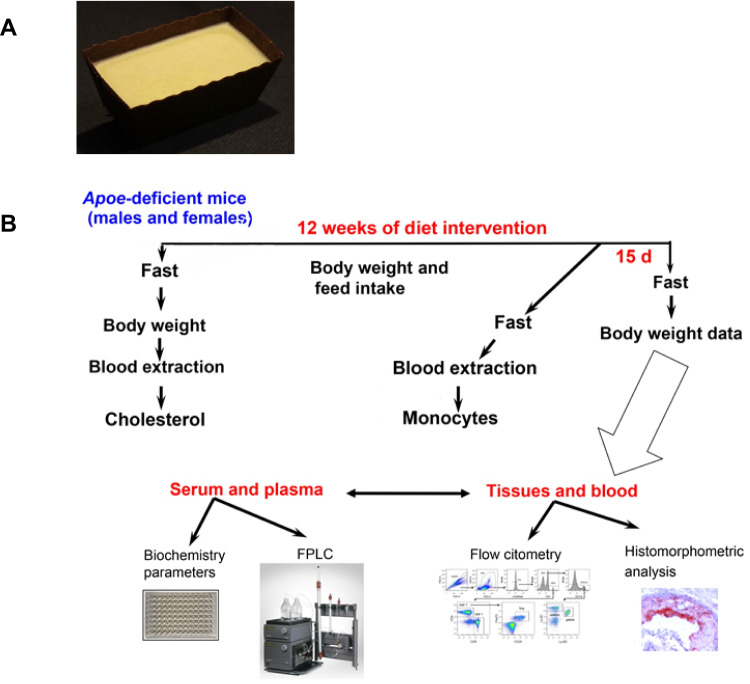
Physical aspect of S-VO (**A**). Experimental design and analytical methods used (**B**)

At the end of the experiment, after 4-h fasting, the mice were weighed and sacrificed by suffocation in a CO_2_ chamber. Blood samples were collected by cardiac puncture, and plasma and serum were centrifuged at 3000 × g for 10 min. Livers were quickly removed, frozen in liquid nitrogen and stored at -80 °C until processing, and an aliquot was stored in buffered formaldehyde for histology. Hearts and aortas were perfused with PBS, hearts were filled with OCT Tissue-Tek® (Sakura Finetek, Barcelona, Spain), frozen in liquid nitrogen and stored at -80 °C, and aortas were dissected and stored in buffered 10% formaldehyde at 4 °C. The experimental design is shown in Fig. 1.

### Diets

During the intervention, the mice were fed with a pelleted Western-style purified diet containing either, 20% S-VO or a commercial edible butter and both supplemented with 0.15% of cholesterol. These diets, differing only in the source of fat and were prepared according to the recommendations of the Nutrient Requirements of Laboratory Animals [39] and their components have been previously described [31]. After preparation, the diets were frozen, lyophilized, and immediately stored at -20 °C in vacuum-sealed bags until use.

### Fatty Acids and Phenolic Compounds Analyses

The fatty acid profile was determined by gas chromatography according to the official method approved by the European Commission [8]. The phenolic extract of solid olive oil was obtained according to the procedure of Montedoro et al. (1992) [32]. The identification and quantification of the individual phenolic compounds were carried out by UPLC-MS/MS on an AcQuity Ultra-Performance™ liquid chromatography/tandem mass spectrometry system (Waters, Milford, MA, USA). The chromatographic conditions were those described by Delpino-Rius et al. [12]. Calibration curves of commercial standards were used to quantify the individual phenolic compounds.

### Plasma parameters

Total plasma cholesterol and triglyceride concentrations were measured in a microtiter assay using commercial Infinity™ kits (Thermo Scientific, Madrid, Spain), glucose (BioSystems, Barcelona, Spain) and HDL cholesterol (HDL-c) according to the Grove protocol [16]. Total serum apolipoprotein A1 (APOA1) and apolipoprotein A4 (APOA4) were quantified by ELISA as previously described [33]. Plasma lipoprotein profile was determined in 100 µL of pooled plasma samples from each group and sex by fast protein liquid chromatography (FPLC) gel filtration using a Superose 6B column (GE Healthcare, Chicago, Il, USA) as previously described [29].

### Evaluation of atherosclerotic lesions

*En face* analyses of dissected aortas and the cross-sectional analyses of aortic roots and aortic lesion characteristics were carried out as previously described [31].

### Hepatic histological analyses

Liver specimens stored in buffered formaldehyde were embedded in paraffin, and Sects. (4 μm) were stained with hematoxylin and eosin. A Zeiss AsioScan.Z1 (Zeiss, Oberkochen, Germany) slide scanner was used to take images of all specimens. Lipid droplets were evaluated by quantifying their areas in each liver section using Adobe Photoshop CS3 (Adobe Inc. San Jose, CA, USA) and expressed as a percentage of the total liver section as previously described [18].

### Analysis of surface molecule expressions in circulating monocytes

The characteristics of blood monocytes were studied as previously described [18] [2]. Fifteen days before the end of the dietary intervention, the mice were fasted overnight and blood samples were collected from the submandibular (facial) vein for analysis of approximately 1 × 10^6^ white blood cells resuspended in PBS.

### Statistical analyses

Data are presented as mean ± SD. Variables that did not show a normal distribution (according to the t student test) or homology of variance were analyzed using the one-tailed Mann–Whitney U test. Statistical analyses were performed with the Statistical Package for Social Sciences version 15 (SPSS, Chicago, IL, USA) or Prism 5 software for Windows (GraphPad, S. Diego, CA, USA). Spearman bivariate correlations were tested between individual data, and GraphPad PRISM® software version 5.02 was used to plot the ROC curve and calculate the area under the curve (AUC) and p value. Differences were considered significant at P < 0.05.

## Results

### Fatty Acids and Phenolic Compounds profiles of S-VO

As shown in Table 1, the S-VO contains a high concentration of oleic acid compatible with a virgin olive oil fatty acid composition [25]. On the other hand, the butter contains a 31.8% of palmitic acid and the saturated/unsaturated ratio was 2.7 vs 0.4 for S-VO. This indicates that S-VO is rich in monounsaturated fats, whereas dairy butter is rich in saturated.

**Table 1:** Fatty acid composition of spreadable virgin olive (S-VO) and butter

Fatty acids	S-VO%	Butter %
Butiric (C4:0)		4
Caproic (C6:0)		2
Caprylic (C8:0)		1
Capric (C17:0)		3.1
Lauric (C12:0)		3.4
Miristic (C14:0)	<0.03	11.2
Pentadecanoic (15:0)		1.2
Palmitic (C16:0)	17	31.8
Palmitoleic C16:1)	1	
Margaric (C17:0)	0.1	
Margaroleic (C17:1)	0.2	
Estearic (C18:0)	11	10.4
Oleic (C18:1)	62	22.6
Linoleic (C18:2)	8	2
Linolenic (C18:3)	0.5	0.35
Araquidic (C20:0)	0.5	
Gadoleic (C20:1)	0.2	
Behenic (C22:0)	0.1	
% SFA	28.6	73.2
% MUFA	63	24.3
% PUFA	8.4	2.5
Saturated/unsaturated ratio	0.4	2.7

Results are expressed as percentage

The abbreviated phenolic composition of S-VO is also shown in Table 2 and the full composition is shown in Table S1. The most abundant phenol in S-VO was hydroxytyrosol with an amount of 3422 μg/kg. Other characteristic phenolics of EVOOs such as luteolin (616 μg/kg), p-coumaric acid (202 μg/kg), ferulic acid (607 μg/kg), and gallic acid (95 μg/kg) were also present in S-VO, consistent with the fingerprint of EVOO.

**Table 2:** Phenolic compounds of spreadable virgin olive (S-VO)

	μ**g/kg of S-VO**
Cyanidin-3-glucoside chloride	56±5
Epicatechin	13 ±22
Ferulic acid	607±13
Gallic acid	95±21
Hesperidin	37 ± 10
Hydroxytyrosol	3422 ± 242
Ideain chloride	62 ± 6
Kaempferol	56 ± 1
Keracyanin chloride	31 ± 3
Luteolin	616 ± 18
Luteolin-7-o-glucoside	18 ± 1
Methylgallate	13 ± 1
Narirutin	15 ± 1
p-Coumaric acid	202 ± 170
Pelargonidin-3-rutinoside chloride	24 ± 2
Quercitrin	10 ± 2
Rutin	8 ± 1
Tangeretin	27 ± 2
Vicenin II	11 ± 2
Vitexin	7 ± 1

Data are means ± SD of triplicate determinations for each compound

### Feed consumption and body weight

**Fig. 2:**
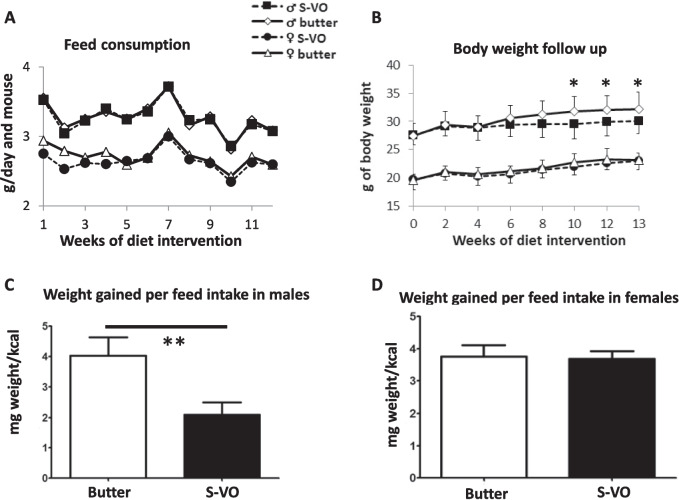
Representative feed consumption (**A**), body weight monitoring during the dietary intervention (**B**). Animal weight gain (mg) in 12 weeks per feed intake (kcal) in males (**C**) and in females (**D**). Results are expressed as mean ± standard deviation. Statistical analysis was performed by Mann–Whitney test or Student's t-test based on normal distribution. *, p ≤ 0.05 butter vs. S-VO and **, p ≤ 0.01 butter vs. S-VO

Feed consumption and body weight follow-up are shown in Fig. 2A and B, respectively. Feed consumption did not show a statistical difference between the two diets, but males showed significant increments in body weight with butter diet starting 10 weeks after the dietary intervention and maintained until the end of the study. When the ratio of body-weight gain to kcal of feed consumption was calculated, the difference between the diets was statistically significant in males, but there was no change in females (Figs. 2C and D).

### Plasma parameters

**Fig. 3:**
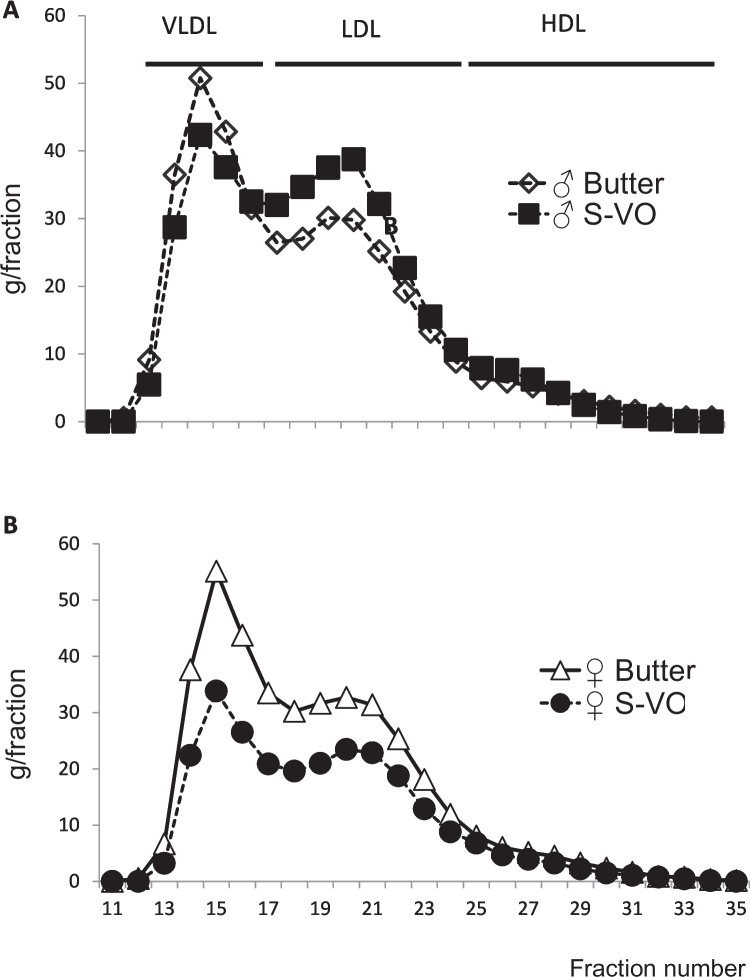
Representative fractions from FPLC profiles. Collected fractions analyzed for total cholesterol in males (**A**) and females (**B**). 13 to 17 fractions corresponded to VLDL, 18 to 24 to LDL, 25 to 29 to cholesterol-rich HDL and 30 to 33 to cholesterol-poor HDL

Plasma analyses showed differences between the two groups (Table 3). In males the butter group reached 796 mg/dL for total cholesterol (TC) and 69 mg/dL for HDL-c, while in the S-VO group the former decreased to 645 and the latter increased to 120 mg/dL, respectively. Despite these remarkable mean values, the changes were not enough to be statistically significant. As shown in the FPLC cholesterol profile in Fig. 3A, the S-VO group had lower VLDL and higher LDL. The increase in HDL-c cannot be observed in this plot. When the TC/HDL-c ratio was calculated, it showed a reduction in males in the S-VO diet without statistical significance (Table 3). In females, plasma analyses showed a significantly lower amount of total cholesterol in the S-VO group, with a reduction from 785 to 699 mg/dL (Table 3). This result was also observed in Fig. 3B, without changes in the lipoprotein profiles, but with a lower amount of cholesterol in the S-VO-fed females. No changes in APOA1 and APOA4 were observed in either sex (Table 3).

**Table 3:** Plasma parameters

	Butter group	S-VO group
Males	(*N* = 14)	(*N* = 14)
Triglycerides (mg/dL)	338 ± 161	340 ± 158
Total cholesterol (mg/dL)	796 ± 263	645 ± 249
HDL colesterol (HDL-c) (mg/dL)	69 ± 36	120 ± 79
Ratio TC/HDL-c	12 ± 5	6 ± 6^a^
APOA1 (arbitrary units)	10 ± 2	10 ± 2
APOA4 (arbitrary units)	6 ± 2	6 ± 2
Females	(*N* = 8)	(*N* = 8)
Triglycerides (mg/dL)	292 ± 91	298 ± 55
Total cholesterol (mg/dL)	785 ± 188	699 ± 77^a^
HDL colesterol (HDL-c) (mg/dL)	65 ± 32	56 ± 17
Ratio TC/HDL-c	12 ± 11	12 ± 6
APOA1 (arbitrary units)	10 ± 1	10 ± 2
APOA4 (arbitrary units)	5 ± 2	5 ± 1

Data are means ± SD for each group. Unless specified, statistical analysis was carried out by Mann Whitney test or test t student based on its normal distribution. ^a^, P < 0.05 vs butter

### Monocyte analyses

Figure S2A and B shows that CD49d + total was higher in males fed with S-VO while CD11b-CD49d- was lower. In females, there were higher statistically significant levels of CD49 high and CD11b + CD49d + in the group fed with S-VO (Figure S2C and D).

### Atherosclerotic lesions

**Fig. 4:**
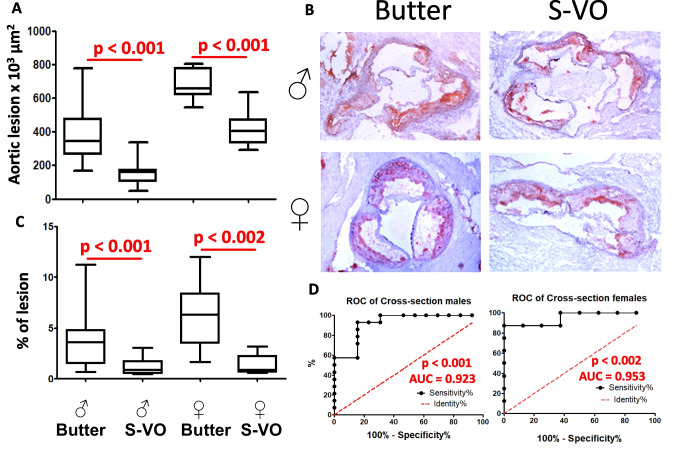
Atherosclerotic lesions in the different experimental groups. Box and whiskers plots show the 5th to 95th percentile of individual aortic cross-sectional analysis (**A**). Representative images of aortic lesions at the valve level representative of each experimental condition (**B**). Presence of atherosclerotic foci as % of lesion *en face* study (**C**). ROC of cross-sectional areas in males and females (**D**). Statistical analyses were performed by Mann–Whitney test or t-Student test based on their normal distribution

Males and females consuming the S-VO-containing diet showed statistically significant lower scores in both the cross-sectional and en face analyses (Fig. 4A and C). In addition, the ROC values of the cross-sectional analyses showed AUC values of 0.923 and 0.953 for males and females respectively (Fig. 4D), indicating good discrimination between groups.

### Correlations among Parameters

**Fig. 5:**
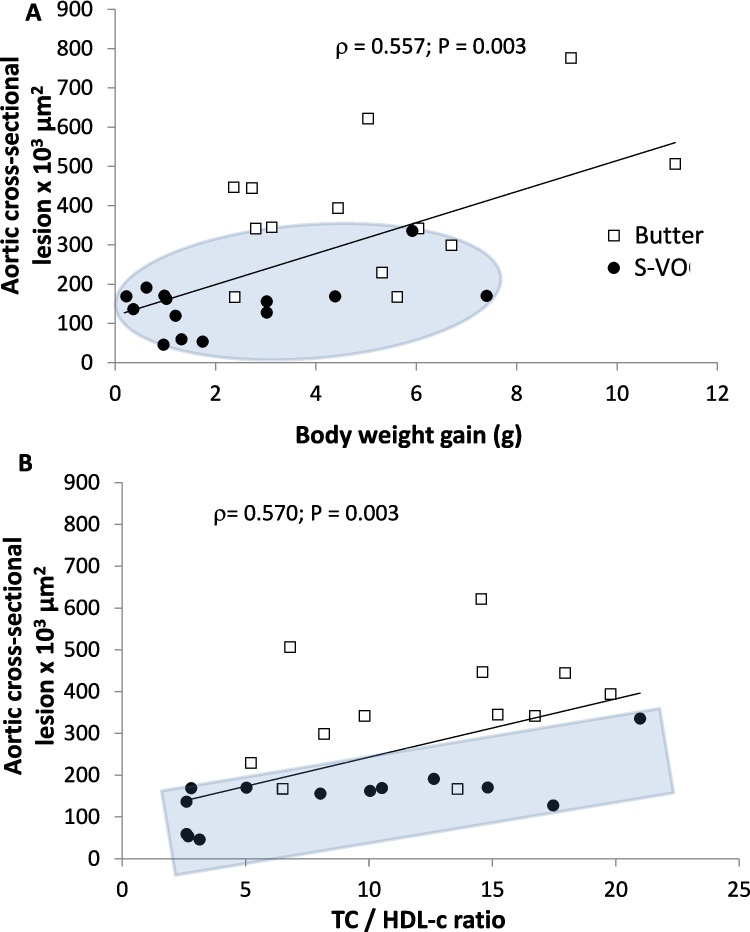
Correlation plots in males. Scatter plot of individual data of aortic atherosclerotic cross-sectional lesion versus body weight gain during the 12-week dietary intervention (**A**), and scatter plot of aortic cross-sectional lesion versus total cholesterol/HDL cholesterol ratio (**B**). Statistical correlations were performed using bilateral Spearman bivariate correlation analysis

According to the obtained correlations, the lower atherosclerotic lesion in S-VO groups in both sexes were associated with different parameters suggesting different mechanisms in males and females. Figure 5 shows the best fits of the results obtained in males. Cross-sectional aortic atherosclerotic lesions were positively associated with body weight gain (Fig. 5A) and with total to HDL cholesterol ratio (Fig. 5B). Interestingly, animals of S-VO group with high TC/HDL-c ratio, showed lesser lesion than animals fed with butter.

**Fig. 6:**
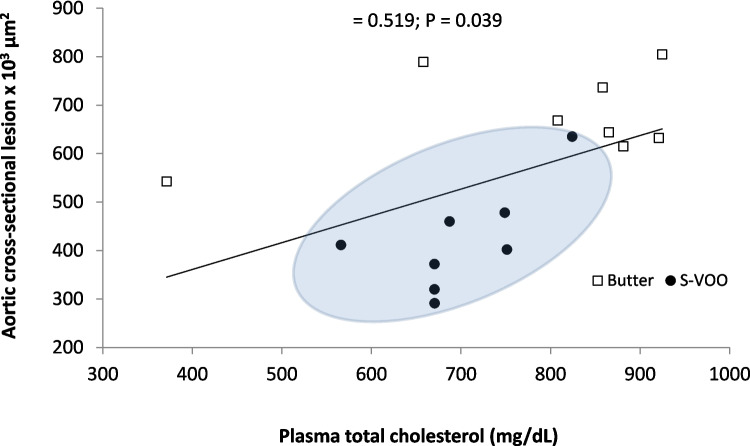
Correlation plots in females. Scatter plot of individual data of aortic atherosclerotic cross-sectional lesion vs. total cholesterol at the end of the 12-week dietary intervention. Statistical correlations were performed with bilateral Spearman bivariate correlation analysis

In females, the aortic atherosclerotic cross-sectional lesion was positively associated with plasma total cholesterol (Fig. 6) and inversely associated with CD49d high (Figure S3A) and with CD11b + CD49d + (Figure S3B). These associations suggest that factors influencing the development of atherosclerosis in this dietary intervention varies between sexes.

## Discussion

The present study was carried out to investigate the anti-atherosclerotic effect of a new formulation of extra virgin olive oil in solid form compared to butter. This new preparation was achieved by mixing cocoa butter with more than 75% of organic EVOO, while still maintaining their high content of oleic acid (62%) and phenolic compounds, characteristic as clear markers of Arbequina VOO origin. Males consuming S-VO showed a lower weight gain at the same feed intake and a lower TC/HDL-c ratio. In contrast, in females, no changes in body weight were observed, but a lower amount of plasma cholesterol was observed under the influence of S-VO. In both sexes, the consumption of S-VO resulted in fewer atherosclerotic lesions than in the butter group. The S-VO diet also influenced the surface expression of circulating monocytes. Correlation studies showed that the aortic root atherosclerotic lesion was associated with sex. Whereas weight gain and the TC/HDL-c ratio were the main factors involved in males, total plasma cholesterol and the profile of circulating monocytes were involved in females. This suggests a sex-specific involvement of lipids and immunological players in the dietary response.

The blend obtained demonstrated the Arbequina source of EVOO, as shown by the high content of linoleic acid and the low content of oleic acid. This variety of EVOO is also rich in palmitic acid compared to others such as Picual, Empeltre or Cornicabra [4]. The high content of stearic acid reflects the contribution of cocoa butter and shows that it was the only fatty acid out of the range corresponding to EVOO (0.5 and 5%) [27]. However, this slight increase in stearic could be metabolized by the stearoyl-CoA desaturase 1, a highly efficient enzyme which transforms stearic into oleic, especially in high fat diet, and therefore reducing reactive oxygen species generating β-oxidation, thus involved in the remodeling of cardiac metabolism and helps to maintain the homeostasis of the cardiovascular system [6]. S-VO showed a value of palmitic acid of 17% of the fatty acid composition, which was in the range of EVOO (7.5 to 20%) [27], and the saturated/unsaturated ratio was of 0.4 versus 2.7 for dairy butter (Table 1).

The phenolic composition of S-VO shows that the hydroxytyrosol content (3,422 μg per kg) was relatively low compared to the values of other EVOO, but in concordance with the characteristics of EVOO obtained from Arbequina variety with low degree of ripeness. It is known that this cultivar is not particularly enriched in hydroxytyrosol compared to others [4] and the early collection of fruits is also a factor modulating its content [41]. On the other hand, the total phenolic analysis, shown in Table S1, evidenced the presence of the main EVOO phenols and it points out that this preparation preserves the phenolic fingerprint of the EVOO used, reinforcing its authenticity. This new formulation raises new interesting concerns to fit in the current categories of the International Olive Oil Council [35]. The compounds of the unsaponifiable fraction are bioactive as demonstrated by Acin et al. [3], who observed changes in hepatic gene expression in mice fed on olive oil enriched in unsaponifiable compounds, using as control the same olive oil with the standard unsaponifiable composition. They showed changes in several biological processes such as fatty acid synthesis, fatty acid transport, lipid metabolism, electron transport, acetyl-CoA biosynthesis, carbohydrate metabolism and glutathione conjugation among others, due only to the compounds of the unsaponifiable fraction of EVOO. Furthermore, several researches have demonstrated complex biological effects of isolated compounds of the unsaponifiable fraction of EVOO such as squalene, hydroxytyrosol, erythrodiol and oleanolic acid among others [1, 2, 14, 38]. In the present work, the presence of unsaponifiable compounds in S-VO (Table 2) in a similar amount to commercial EVOO [27] compared to an animal fat deprived of them could contribute to the beneficial effects observed in the S-VO and supply in solid format.

To study the effects of this new S-VO formulation on atherosclerosis and fatty liver, the *Apoe*-deficient mouse was used since it has been proved to be a consistent animal model in dietary interventions of those pathologies [19, 30]. When somatometric parameters were followed and despite the same feed intake, there were statistical significances in body weight of males consuming the S-VO vs butter, a finding that took place 10 weeks after the beginning of the intervention (Fig. 2B). These results are in line with those of Havranek et al. who observed that the Mediterranean diet was more effective than a low-fat diet for reducing body weight, body mass index, and blood pressure in overweight patients with increased cardiovascular risk [20]. De la Puebla et al. using isocaloric diets reported that the substitution of a saturated fat-rich diet by a Mediterranean or carbohydrate-rich diet decreased total body fat in hypercholesterolemic male subjects [9]. These results in humans reinforce the value of *Apoe*-deficient mice to study human diseases as such as those associated with overweight. Another possibility for the body weight changes in males could be due to the fatty acid composition of the two sources of fats used. As mentioned in the results, S-VO is an unsaturated fat, whereas butter is saturated (Table 1). It could be that the greater amount of palmitic acid content of the dairy butter groups and may lead to protein palmitoylation, one of the most important post-transcriptional modifications (PTMs) involved in the regulation of protein signaling, trafficking, localization and enzymatic activities in various tissues and cells, which could modify the regulation of protein function [24, 28]. Ren et al. discover that palmitoylation is involved in protein trafficking membrane with modifications in proteins expressed in adipocytes, playing a role in lipid storage and glucose homeostasis, which could explain the statistical significance in body weight in males without changes in feed consumption [40]. Palmitoylation of endothelial nitric oxide synthase (eNOS) decreases nitric oxide production, while insulin resistance and fatty acid synthase downregulation [28, 46], implying a reduction of de novo lipogenesis mediated by MyD88 palmitoylation as observed in non-alcoholic steatohepatitis treatment with caffeine, which could justify the differences in body weight in males [44]. This result was in agreement with Kwak et al. who showed that palmitic acid induces ER stress, oxidative stress and insulin resistance via AMPK activation in liver and adipose tissue and induces lipotoxicity with ER stress, inflammation and insulin resistance in skeletal muscle [23]. Peroxiredoxin 6 (PRDX6) is a member of the thiol-specific antioxidant protein family with phopholipase A_2_ activity, and the palmitoylation status could modify the cellular redox status in a glutathione-dependent manner by transferring palmitate to glutathione through depalmitoylation of PRDX6, and could be another mechanism for modulating cellular redox status [21]. In addition, our research group have recently shown that the endoplasmic reticulum protein TXNDC5 interacts with PRDX6 to modulate the lipid peroxidation system and the glutathione mechanism in AML12 cells which may be disrupted by the absence of TXNDC5, a novel protein–protein interacting partner of PRDX6 and HSPA9 [7]. The interaction of palmitic acid with these proteins seems a promising field of study.

Plasma analyses revealed differences in the handling of cholesterol among lipoproteins in both sexes. In males, the TC/HDL-c ratio was significantly lower in the animals consuming the S-VO compared to the butter group. This result indicates that in males consuming the S-VO more cholesterol was vehicle in HDL and therefore the ratio decreased. Considering the absence of changes in APOA1, the main protein of HDL [25, 26], it would suggest a greater size of HDL particles. This could explain the increase in fractions 18 to 24, which theoretically correspond to LDL (Fig. 3 A), although they also coelute with large APOE HDL [10]. Since the experiments were performed in *Apoe*-deficient mice, the large HDL cannot be those containing APOE. In females, there was a statistically significant decrease in total cholesterol in the S-VO group compared to the butter group. A decrease that corresponded to a decrease in VLDL and LDL particles (Fig. 3B). This clear reduction in lipoproteins was previously observed in female mice consuming Arbequina EVOO [4]. These results clearly highlight the influence of sex on the lipoprotein response to VOO in a hypercholesterolemic model fed a Western diet.

Two surface proteins of circulating monocytes, ITGA4 (CD49d) and Mac-1 (CD11b), were analyzed by flow cytometry. The former is involved in cell adhesion to fibronectin, vascular cell adhesion molecule 1 (VCAM-1) and intercellular leukocyte interactions [37], while the latter is a proinflammatory member of the CD18 family of leukocyte adhesion receptors involved in autoimmune diseases [42]. As shown in Figure S3, the population expressing both proteins changed according to sex and diet. Only in females, the presence of both proteins was increased by S-VO administration and was paradoxically inversely associated with the development of cross-sectional aortic root lesions (Figure S3A and B). These results demonstrate the influence of sex on inflammatory markers and their response and also emphasize that in some specific dietary settings, they may not be simple surrogate markers of the atherosclerotic process to which they may contribute.

The atherosclerotic lesions examined in cross-sectional aortic roots and en face dissection of the entire aortic tree showed a lower lesion in the S-VO in both sexes and in both analyses (Fig. 4A, B and C). These two atherosclerotic assessments suggest that the growth of established atherosclerotic lesions, as measured by the cross-sectional procedure, and the presence of new atherosclerotic foci along the aortic tree, as measured by en face analyses, are favorably influenced by the administration of S-VO. Furthermore, the ROC analyses and the high AUC values of 0.923 and 0.953 obtained in males and females, respectively, indicate a very uniform response to perfectly discriminated groups. These results extend previous findings of lower lesions in female *Apoe*-deficient mice consuming EVOO from different cultivars compared to palm oil [4]. Once again, the comparison of a saturated fat, in this case of animal origin, resulted in a worse outcome than the mixture of S-VO in Western diets. Interestingly, a certain content of palmitic and stearic acid in the setting of unsaponifiable of VOO may be tolerable regarding the development of atherosclerosis and opens a field for the development of new blends.

In this experiment, no changes in hepatic steatosis were observed between the two different diets used in either sex, as shown in Figure S1. This is in contrast to our previous result using a similar percentage of fat as EVOO in female *Apoe*-deficient mice [5]. Several factors should be taken into account, as in the previous paper EVOO was compared to palm oil, the diets were not purified and the mixed genetic background of Ola129xC57BL/6 J mice was used. The latter was shown to be an important contributor to the assessment of hepatic lipid droplet areas [43]. As C57BL/6 J mice develop a fattier liver, the chosen experimental setting might not be sensitive enough to assess pathological differences between these two diets.

Correlation studies were performed to determine the contribution of different parameters to atherosclerotic development. These results clearly indicate a sex difference in the effect of S-VO. In males, two parameters, body weight gain and the TC/HDL-c ratio, showed the strongest correlation values with atherosclerotic lesions in the aortic root (Fig. 5), and the populations of mice consuming the different diets were clearly differentiated. In females, total cholesterol was directly correlated with atherosclerotic lesion (Fig. 6) and monocyte surface markers showed an inverse association (Figure S3). These findings suggest sex differences in the mechanisms of dietary response.

In conclusion, the mechanisms of dietary response to the progression of atherosclerosis are sex-dependent. Despite this observation, the consumption of S-VO resulted in a lower growth of established plaques and a lower number of new atherosclerotic foci in both sexes compared to mice consuming butter. From a nutritional point of view, the amount of palmitic acid and stearic acid that can be included in the unsaponifiable VO setting needs to be tested in terms of atherosclerosis development and to open a field for the development of new blends.

## Supplementary Information

Below is the link to the electronic supplementary material.

Supplementary file1 (DOC 610 KB)

## Data Availability

Data will made available to scientists on reasonable request.
