# Supplementary material for: Effect of virgin olive oil as spreadable preparation on atherosclerosis compared to dairy butter in Apoe-deficient mice
Source: J Physiol Biochem. 2024 May 24;80(3):671–83. doi: 10.1007/s13105-024-01029-8 (PMC11502577; doi:10.1007/s13105-024-01029-8)

Table S1. Total phenolic composition of S-VO.

| **Compound** | **μg/kg of S-VO** |
| --- | --- |
| 3.5 dicaffeoyl quinic acid | <1 |
| Arbutin | <1 |
| Caffeic acid | <1 |
| Catechin | <1 |
| Chlorogenic acid | <1 |
| Cyanidin-3-glucoside chloride | 56 ± 5  |
| Daidzein | <1 |
| Delphinidin-3-o-rutinoside chloride | <1 |
| Ellagic acid | <1 |
| Epicatechin | 13 ± 22 |
| Epicatechin gallate | <1 |
| Epigallocatechin gallate | <1 |
| Eriocitrin | <1 |
| Eriodictyol | <1 |
| Ferulic acid | 607 ± 13 |
| Fisetin | <1 |
| Fumaric acid | <1 |
| Galangin | <1 |
| Gallic acid | 95 ± 21 |
| Gallocatechin gallate | <1 |
| Genistein | <1 |
| Hesperidin | 37 ± 10 |
| Hydroxytyrosol | 3422 ± 242 |
| Hyperoside + quercetin-3-o-glucopyranoside | <1 |
| Ideain chloride | 62 ± 6 |
| Isorhamnetin-3-o-glucoside | <1 |
| Kaempferol | 56 ± 1 |
| Keracyanin chloride | 31 ± 3 |
| Luteolin | 616 ± 18 |
| Luteolin-7-o-glucoside | 18 ± 1  |
| Methylgallate | 13 ± 1 |
| Myricetin | <1 |
| Narcissoside | <1 |
| Naringin | <1 |
| Naringin dihydrochalcone | <1 |
| Narirutin | 15 ± 1 |
| Oleuropein | <1 |
| Orientin | <1 |
| o-Salicylic acid | <1 |
| p-Coumaric acid | 202 ± 170 |
| Pelargonidin-3-rutinoside chloride | 24 ± 2 |
| Phloretin | <1 |
| Phloridzin | <1 |
| Procyanidin A2 | <1 |
| Procyanidin B1 | <1 |
| Procyanidin B2 | <1 |
| Procyanidin C1 | <1 |
| p-salicylic acid | <1 |
| Quercetagetin | <1 |
| Quercetin | <1 |
| Quercitrin | 10 ± 2 |
| Rosmarinic acid | <1 |
| Rutin | 8 ± 1 |
| Syringic acid | <1 |
| Tangeretin | 27 ± 2 |
| Taxifolin | <1 |
| Vanillic acid | <1 |
| Vicenin II | 11 ± 2 |
| Vitexin | 7 ± 1 |

Data are means ± SD of triplicate determinations for each compound.

Figure S1. Percentage of lipid droplets in the liver at the end of diet interventions.


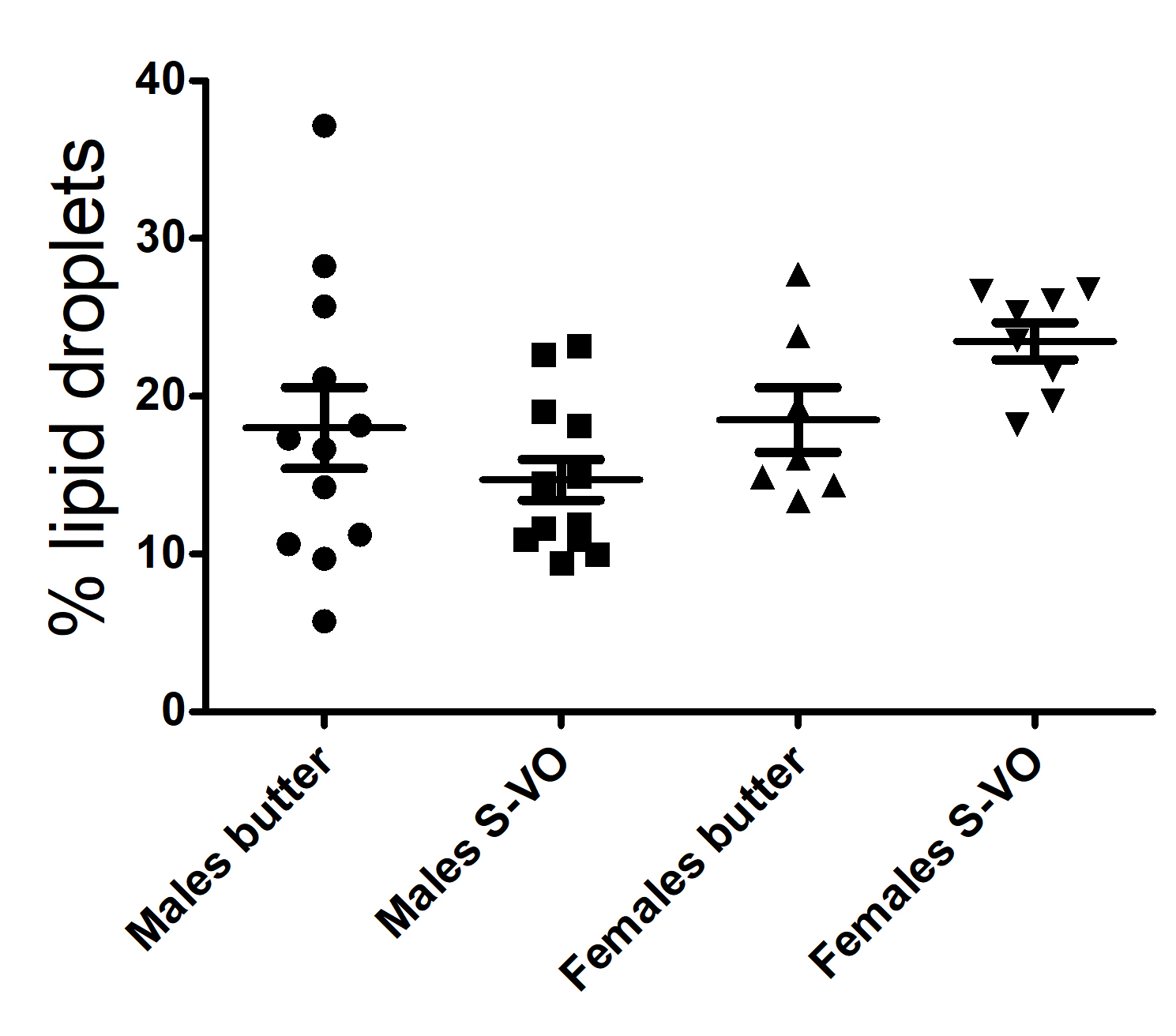


♂ butter ♂ S-VO ♀ butter ♀ S-VO

Scatter plot with the individual data and means ± SD expressed as % of area occupied by lipid droplets.

**Figure S2.** Analysis of surface molecule expressions in monocytes. Approximately 1 x 10^6^ white blood cells samples for each animal were analyzed. Flow cytometry was analyzed for CD49d+total (A), CD11b – CD49d – (B), CD49d high, (C) and CD11b+ Cd49d+ (D). Statistical analyses were carried out by Mann Whitney’s U test or test t student based on their normal distribution.
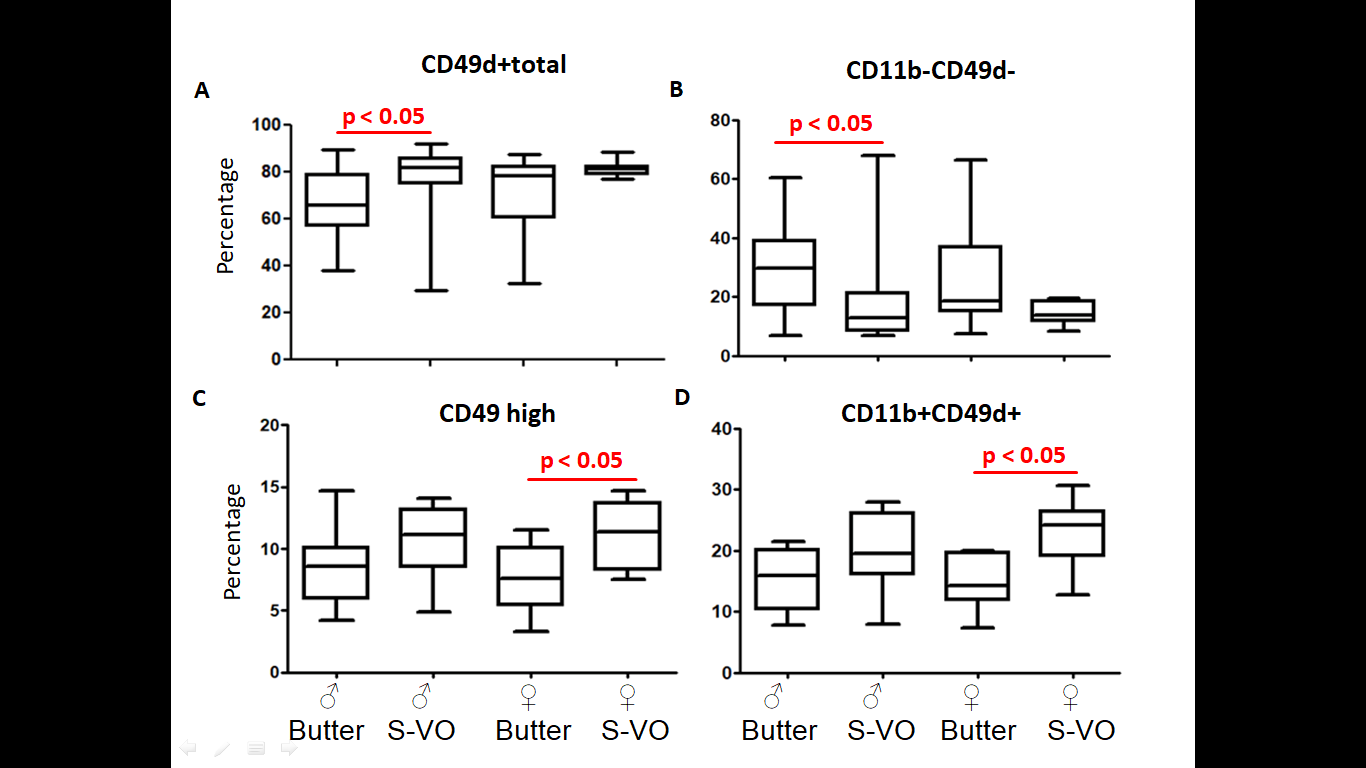


**Figure S3.** Correlation graphs in females. Scatter plot of individual data of aortic atherosclerotic cross-sectional lesion vs CD49d high (A) and vs CD11b + and CD49d + (B). Statistical correlations were carried out with bilateral Spearman bivariate correlation analysis.


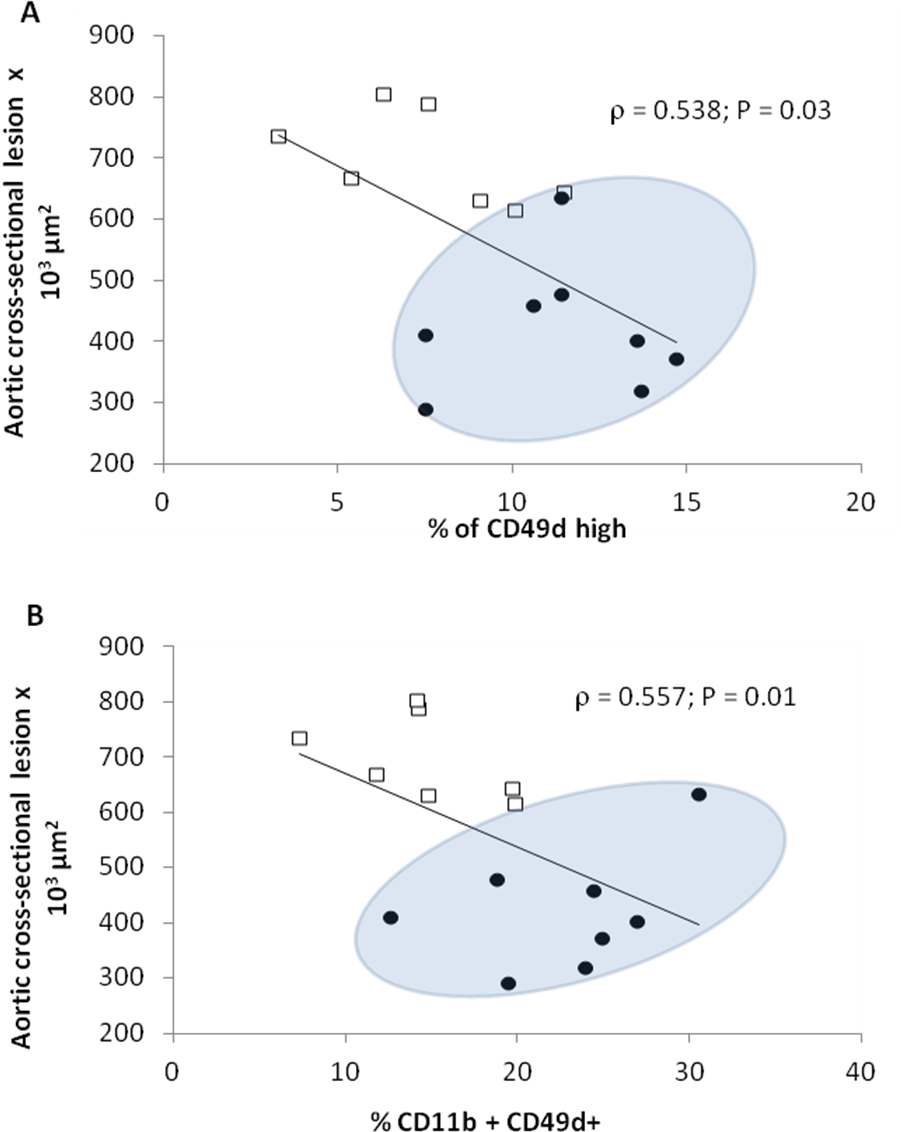

Supplement: Supplementary file 1 — Supplementary file1 (DOC 610 KB) [file 13105_2024_1029_MOESM1_ESM.doc]
